# Phytochemical Profile and In Vitro Bioactivities of Wild *Asparagus stipularis*

**DOI:** 10.3390/molecules29040817

**Published:** 2024-02-10

**Authors:** Amel Hamdi, Sara Jaramillo-Carmona, Rocío Rodríguez-Arcos, Ana Jiménez-Araujo, Najoua Karray Bouraoui, Rafael Guillén-Bejarano

**Affiliations:** 1 Phytochemicals and Food Quality Group, Instituto de la Grasa (CSIC), 41013 Seville, Spain; smjaramillo@ig.csic.es (S.J.-C.); rrodri@ig.csic.es (R.R.-A.); araujo@ig.csic.es (A.J.-A.); 2 Unité de Physiologie et de Biochimie de la Réponse des Plantes aux Contraintes Abiotiques, Faculté des Sciences de Tunis, Université Tunis El Manar, Tunis 2092, Tunisia; najouakarraybouraoui@yahoo.fr

**Keywords:** *A. stipularis*, saponins, phenolic acid, flavonoids glycoside, pancreatic lipase inhibitory, antioxidant activities, cytotoxic effects, LC-MS, HPLC-DAD, HPLC/Q-TOF-MS

## Abstract

In this study, *Asparagus stipularis* was characterized concerning its phytochemical composition, antioxidant potential, cytotoxicity, and pancreatic lipase inhibitory activities. Twenty-seven compounds were identified and quantified by HPLC-DAD-MS in the leaf, stem, pericarp, and rhizome of ethanolic extracts. Seven steroidal saponins were detected, and the highest content was quantified in rhizome and pericap. *A. stipularis* also contained significant amounts of flavonoids in the aerial part. Isorhamnetin tetra-glycoside, quercetin-3-glucosyl-rutinoside, and rutin were the main flavonoid derivatives in leaf, stem, and pericarp extracts, respectively. In addition, eleven phenolic acids were also detected; among them, caffeic acid, protocatechuic acid, p-hydroxybenzoic acid, and ferulic acid were the predominant phenolics, with these having the highest amounts quantified in the rhizome extracts. All the tested extracts possessed antioxidant capacities, with pericarp and rhizome extracts exhibiting the highest activity in DPPH, ABTS, and FRAP assays. The extracts from pericarp and rhizome were revealed to also be the strongest inhibitors of pancreatic lipase. The rhizome extracts exhibited potent cytotoxic activity against HCT-116 and HepG2 with IC50 values of 30 and 54 µg/mL after 48 h of treatment. The present study demonstrated that *A. stipularis* can be used as a new source of natural antioxidants and potential anticancer and antiobesity compounds.

## 1. Introduction

Numerous wild plants have the potential to serve as sources of both food and medicine, making them cost-effective, locally available, and versatile resources that can enhance the nutrition and health of populations [1]. Valuable wild plants can be found in various regions across the globe, and many of them play a role in herbal therapy, which is deeply ingrained in the culture and traditions of different communities. Moreover, a significant number of phytochemicals found in wild plants have been associated with the prevention of specific diseases [2]. Identifying these active compounds and understanding their mechanisms of action is of paramount importance. Therefore, comprehensive research involving both plant extracts and the individual active compounds they contain is essential.

*Asparagus* has played a prominent role in the history of food and agriculture, spanning from ancient times to the present day. In contemporary agriculture, many species of *Asparagus* are cultivated for commercial purposes, with the most economically significant being garden asparagus, scientifically known as *A. officinalis*. Like other vegetable crops, *A. officinalis* faces various agricultural challenges such as drought, crop failure, and pest infestations [3]. Interestingly, certain wild *Asparagus* species, including *A. acutifolius*, *A. albus*, *A. stipularis*, and *A. maritimus*, exhibit resistance or tolerance to these factors. Moreover, these wild species have a long history of traditional medicinal use, with preparations ranging from medicinal infusions and decoctions to macerations, syrups, poultices, broths, or direct applications for the treatment of various health disorders.

Prior research has highlighted the nutritional richness of wild *Asparagus*, particularly *A. acutifolius* [4], *A. albus* [5], and *A. stipularis* [6], which are abundant sources of essential minerals, dietary fibers, vitamins, and amino acids. Furthermore, these wild varieties are noteworthy for their high content of bioactive compounds, primarily simple phenolics, flavonoids, and saponins, which are closely linked to their functional properties [4,5,6,7].

Most research on the bioactive properties of *Asparagus* has been carried out on extracts obtained from the shoots of *A. officinalis* and the roots of *A. racemosus* [8,9]. Different parts of *A. racemosus* and *A. albus* have been suggested to have health benefits, such as improvement in the immune system and cancer prevention [5,6,7,8,9,10,11].

*A. stipularis Forssk*. Is an *Asparagaceae* plant, commonly known in Egypt, Algeria, and Tunisia [6,7,8,9,10,11,12,13]. Infusion from the tuberous roots of the plant is used in the Mediterranean area to remove renal stones, cure syphilis, and treat headache. The decoction of a whole plant is used to relieve stomach ache and promote appetite, having an effect that is similar to that obtained from the consumption of berries. Shoots and roots are used as diuretics and also for the prevention or treatment of jaundice, liver ailments, bilharzias, and rheumatism. It is also believed that plants fried with eggs and camel’s fat stimulates spermatogenesis, facilitates secretions, and releases obstructions. On the other hand, seeds are decocted for hemorrhoids [14]. Regarding specific components, recent studies have shown that the saponins from *A. stipularis* roots exhibit anti-inflammatory [15] and antischistosomiasis activities [13]. Moreover, Adouni et al. [6] reported that *A. stipularis* might be an interesting candidate to improve and recover wild plant consumption, including its young shoots, as part of the Mediterranean diet within so-called modern diets rich in antioxidant and biofunctional foods and food products. This vegetable also has potential for the development of new antioxidant and cancer chemopreventive dietary supplements or nutraceuticals.

This study seeks to characterize bioactive compounds from *A. stipularis* leaf, stem, pericarp, and rhizome, with a specific focus on functional properties such as antioxidant, cytotoxic, and lipase inhibitory activities within hydro-alcoholic extracts.

## 2. Results and Discussion

### 2.1. Analysis of Simple Phenolics

The analysis of ethanolic extracts from *A. stipularis* using HPLC-DAD-MS revealed the presence of eleven distinct simple phenolics and phenolic acids. These compounds were identified by comparing their retention times, absorption spectra, and mass fragmentation patterns with data obtained from commercial standards. The quantification of each compound was performed by comparing peak areas from the HPLC-DAD chromatogram with calibration curves derived from corresponding standards. The results are presented in Table 1.

As observed, the phenolic composition and content of extracts from leaves, stems, pericarps, and rhizomes were significantly different. The highest total phenolics content was measured in rhizome extracts, reaching 930 mg/kg DW, while the contents from the pericarp and stem were 290 and 219 mg/kg DW, respectively. The lowest amount was found in the leaves (122 mg/kg DW). These results contrast with the findings reported by several other authors, who noted that the highest phenolic contents were typically found in the reproductive and vegetative organs [16,17,18,19].

Caffeic acid was identified as the most abundant phenolic acid in both leaf and stem extracts, constituting 26% and 43% of the total phenolic complement, respectively. On the other hand, protocatechuic acid emerged as the predominant phenol in the pericarp, reaching a concentration that represented 60% of the total phenolics. The phenolic composition of rhizome extracts displayed a greater variety of compounds, with hydroxybenzoic acid and ferulic acid being the most abundant, each accounting for 17% of the total phenolic content.

In addition to the major phenolics identified in various organs, a significant amount of ferulic acid (21%) and hydroxybenzoic acid (19%) was also detected in the leaves. However, in stem and pericarp extracts, the second major phenolic acids were gallic acid (16% and 13%) and ferulic acid (13% and 8%), respectively. Protocatechuic acid (11%) and coumaric acid (14%) were found in significant amounts in rhizome extracts. Vanillic acid, vanillin, and sinapic acid were also detected in *A. stipularis* organs, albeit in very low amounts, ranging from 1% to 9%.

This study highlights the well-established differences in the accumulation of phenolic compounds among distinct organs of medicinal plants [20]. Furthermore, it emphasizes that concentrations of phenolics not only vary from plant to plant but also within different parts of the same plant species [21].

### 2.2. Identification of Flavonoid Glycosides in Different Asparagus Parts

The analysis of the ethanolic extract obtained from various parts of *A. stipularis*, including the leaf, stem, pericarp, and rhizome, revealed the presence of six flavanol glycosides and three flavanols in these extracts. The data, including retention time, maximum absorption in the UV-VIS region, molecular ion, and main fragment ions observed in HPLC-DAD-MS analysis, are provided in Table 2 for reference.

Peaks 3, 4, and 5 correspond to three flavonoid triglycerides previously described in *Triguero* asparagus [22,23,24] and *A. albus* [5]. Peak 6 corresponds to rutin, and peaks 7, 8, and 9 represent three different free aglycones. Peaks 1 and 2, on the other hand, were detected in the chromatogram of the ethanolic extract from leaves and stems. These compounds, not previously identified in asparagus extracts, were tentatively identified as flavonoid glycosides based on their UV spectra, which were similar to those of rutin (Quercetin-3-*O*-rhamnoglucoside).

**Figure 1:**
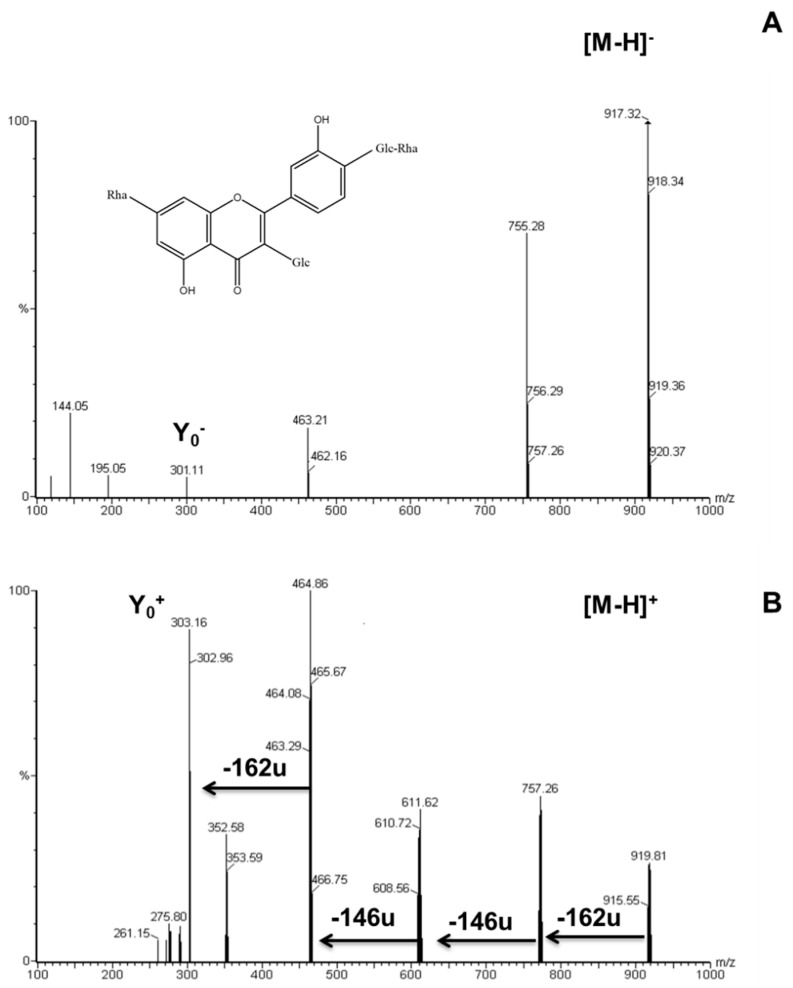
ESI spectra of quercetin tetraglycoside in negative (**A**) and positive (**B**) mode.

The [M − H]^−^ spectrum of compound 1 exhibited a deprotonated molecular ion at m/z 917 and an ion at m/z 301 corresponding to the deprotonated aglycone (quercetin) (Figure 1A). Complementary information from the positive ion spectrum revealed that the fragmentation pattern for the compound ([M + H]^+^, m/z 919) indicated an O-glycoside of quercetin. Data from the positive ion spectrum suggested that this flavonoid derivative contained a hexose, likely glucose (162 u), the loss of which resulted in the ion at m/z 757. The ion at m/z 757 further decomposed into another prominent ion at m/z 611, arising from the loss of a second unit of deoxyhexose, possibly rhamnose (146 u). Subsequently, the ion at m/z 611 decomposed into another prominent ion at m/z 465, resulting from the loss of a second unit of rhamnose (146 u). Finally, the loss of a glucose residue (162 u) generated the major ion at m/z 303, corresponding to protonated aglycone (quercetin) (Figure 1B).

These results suggest that this compound is quercetin-tetraglycoside (Quercetin 3-glucosyl-rhamnosyl-rutinoside). This flavonol tetraglycoside with ([M + H]^+^, m/z 919) had not been previously described in asparagus but had already been identified in other plants, such as *Myrsine africana* leaves [25]. Krasteva et al. [26] demonstrated that in the model of t-BuOOH-induced oxidative stress, quercetin-tetraglycoside, flavonoids isolated from *Astragalus monspessulanus*, had significant hepatoprotective and antioxidant activities, similar to those of silymarin. The cytoprotective effects of these compounds were possibly related to their activity as scavengers of reactive oxygen species (ROS), with effects comparable to those of silymarin, a well-known ROS scavenger.

**Figure 2:**
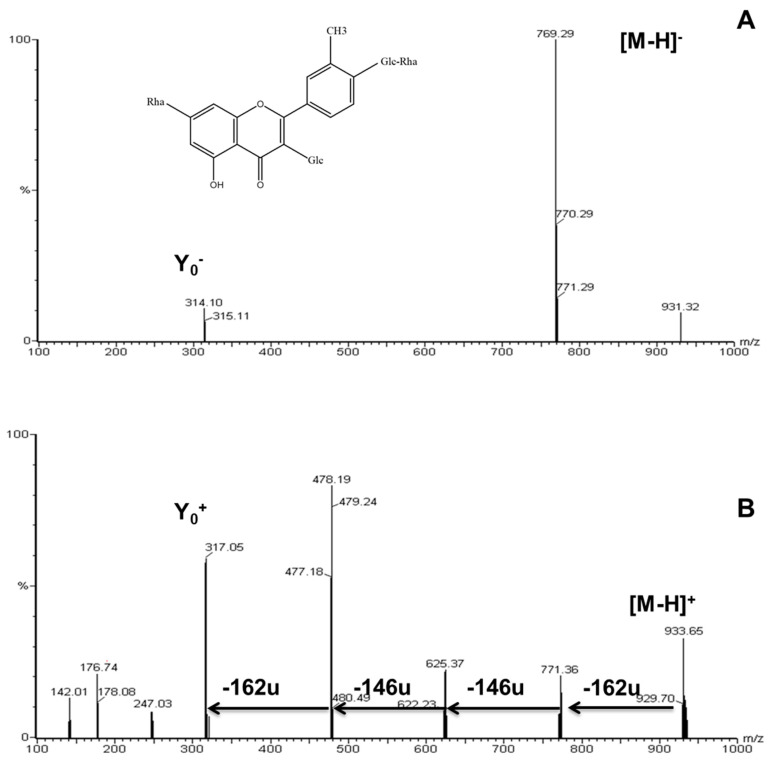
ESI spectra of isorhamnetin tetraglycoside in negative (**A**) and positive (**B**) mode.

The [M − H]^−^ spectrum of compound **2** (Figure 2A) showed a deprotonated molecular ion at m/z 931 and an ion at m/z 315 corresponding to the deprotonated aglycone (isorhamnetin). Complementary information from the positive ion spectrum revealed a fragmentation pattern similar to that of compound **5** (isorhamnetin-3-rhamnosyl-rutinoside), but with the addition of a fourth sugar moiety (162 u). Compound **2** was tentatively identified as isorhamnetin-tetraglycoside (isorhamnetin 3-glucosyl-rhamnosyl-rutinoside) (Figure 2B). To the best of our knowledge, this is the first description of this IR-tetraglycoside in *Asparagus* or any other plant. Given the subsequent results and considering that this IR-tetraglycoside is the predominant flavonoid in the leaves of *A. stipularis*, this plant could be deemed a valuable source of flavonoids with potential pharmaceutical interest.

**Figure 3:**
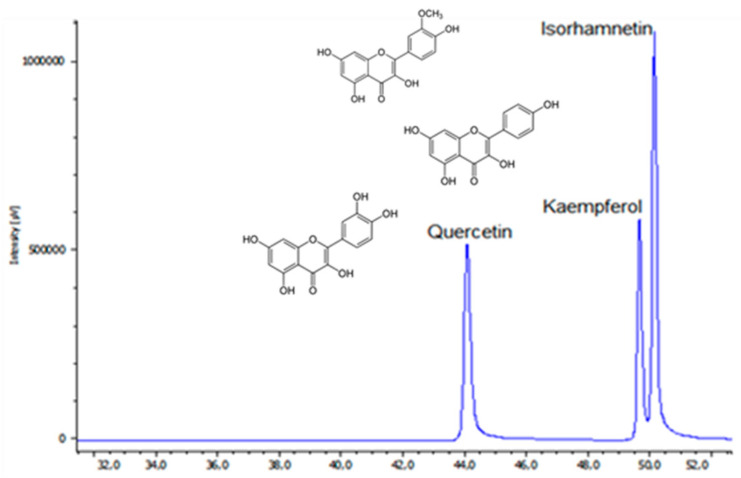
HPLC-DAD chromatogram at 370 nm of the hydrolysate extract of *A. stipularis* leaf and stem.

The acid hydrolysis of the extracts resulted in the isolation of three different flavonols. Figure 3 illustrates that among these flavonol aglycones, isorhamnetin was the most prominent compound found in the extracts from *A. stipularis*. This finding is significant as it distinguishes *A. stipularis* from many other *Asparagus* species, both wild and cultivated, where quercetin glycosides, primarily rutin, are the predominant components and, in some cases, the sole flavonoids present in asparagus tissues [22,23]. The presence of isorhamnetin as the primary aglycone in *A. stipularis* indicates a unique flavonoid profile for this species, which can have implications for its potential uses and medicinal properties. This distinction underscores the importance of studying the chemical composition of different plant species to better understand their potential benefits and applications.

The quantitative results of the different flavonoids are presented in Table 3; the results showed that the total content of flavonoids in the leaf and stem are 2.3 and 1.3 times higher than in the pericarp, respectively.

Six flavonoids were detected in the leaf, four in the stem, and three in the pericarp. Among these compounds, two of them (Q-*O*-tetraglycoside and IR-*O*-tetraglycoside) have been detected for the first time in asparagus, as explained above. They were present in significant quantities in both leaf (14% and 36%) and stem (11% and 26%), respectively. Q-3-*O*-rhamnosyl-rutinoside was identified in the different parts of *A. stipularis* and represented 17% in the leaf, 12% in the stem, and 23% in the pericarp. Q-3-*O*-glucosyl-rutinoside was found in the leaf and stem but not in the pericarp. IR-3-*O*-glucosyl-rutinoside was present in significant amounts in the leaf but was not detected in the stem and pericarp.

Regarding the free aglycones, minimal amounts of naringenin and kaempferol were detected in the leaf (5 and 48 mg/kg DW), stem (6 and 34 mg/kg DW), and pericarp (6 and 70 mg/kg DW), respectively, but not in the rhizome extracts. Quercetin, however, was exclusively identified in the pericarp, with an amount of 44 mg/kg DW.

Notably, significant variations in flavonoid content were observed among leaves, pericarps, and stems of the same species. These findings suggest that, within each plant, the production of flavonoids is independently regulated in each organ. Indeed, the autonomous accumulation of flavonoids in different plant parts has been previously documented as a qualitative or discrete trait [27,28,29].

The flavonoid composition of *A. stipularis*, as described above, differs from that found in our earlier study on *A. albus* [5], where rutin was identified as the major flavonoid in various plant parts. In contrast, in *A. stipularis*, IR-*O*-tetraglycoside emerges as the predominant flavonoid, constituting 36% of the total content in leaf extracts. Rutin, on the other hand, takes precedence in the pericarp (58%).

### 2.3. Analysis of Saponins in Asparagus stipularis Samples

The base peak ion chromatograms of the saponin extracts from *A. stipularis* identified by (UHPLC/Q-TOF-MS (+/−)) are shown in Table 4.

Based on the exact mass, fragment ion analysis, retention times, and structures reported in the literature, seven saponins were tentatively identified from the extract of *A. stipularis*. Notably, four of these compounds (STIPSAP-1, STIPSAP-2, STIPSAP-3, and STIPSAP-4) were identified for the first time in *A. stipularis*, representing novel structures. The predominant saponin across leaf, stem, and pericarp extracts was STIPSAP-2, a compound not previously described in the context of *Asparagus*. Its tentative identification was based on a combination of retention times (Rts) and mass spectra obtained through LC-MS analysis.

In contrast, the main saponin detected in the rhizome was WSAP-4, which has also been noted as the most abundant saponin in *A. maritimus* from Venice, *A. pseudoscaber*, and *A. brachiphyllus* [30]. Additionally, a notable quantity of HTSAP-2 was observed in the pericarp and rhizome of *A. stipularis*. The profile of HTSAP-2 in these parts coincides with that found in several genotypes of green *triguero* asparagus from Huétor-Tájar (Granada, Spain), as reported by Vázquez-Castilla et al. [31].

The mass spectra of STIPSAP-1 (Table 4) in both negative and positive modes are consistent with a saponin containing two pentoses and two hexoses. In negative mode, Table 4 displays the product ions derived from the molecular ion [M − H]^−^ (m/z 1019), showing the loss of a pentose at m/z 887. The ion at m/z 755 originates from the loss of a pentose from the ion at m/z 887. Additionally, the ion at m/z 593 arises from the loss of a hexose, and the ion at m/z 431 (deprotonated genin) results from the loss of another hexose. In the positive spectrum mode, the spectrum reveals ions at m/z 1043 (an adduct formed with sodium), m/z 1003 (loss of one H_2_O molecule), and m/z 871, 709, 577, and 415 corresponding to the sequential loss of a pentose, a hexose, a pentose, and a hexose, respectively.

STIPSAP-2 and STIPSAP-3 appear to be two isomers with the same molecular weight and fragmentation pathway model, but they exhibit different retention times. STIPSAP-2 is the predominant saponin in leaf, stem, and pericarp extracts of *A. stipularis*, while STIPSAP-3 represents approximately 10% of the total saponin content in these organs. The mass spectrum of both STIPSAP-2 and STIPSAP-3 in negative mode showed no fragmentation ions. However, in the positive spectrum mode, the ions at m/z 859 (adduct formed with sodium), m/z 819 (loss of one H_2_O molecule), and m/z 657, 511, and 415 were observed, corresponding to the sequential loss of a hexose, a deoxyhexose, and a unit of 96, respectively (refer to Table 4).

STIPSAP-4, observed in negative-ion mode, displayed a prominent ion peak at m/z 841 [M − H]^−^, indicating a molecular weight of 842. Additional fragment ion peaks were noted at m/z 709, 577, and 433, corresponding to the loss of two pentosyl moieties and 144 Da (C_8_H_16_O_2_). In positive mode, the spectrum revealed a molecular ion peak at m/z 895 [M + Na]^+^ and 825 [-Na-H_2_O]^+^. Other fragment ion peaks were also observed at m/z 693, 561, and 417 (protonated aglycone), corresponding to the loss of a pentose and 144 Da (C_8_H_16_O_2_).

**Figure 4:**
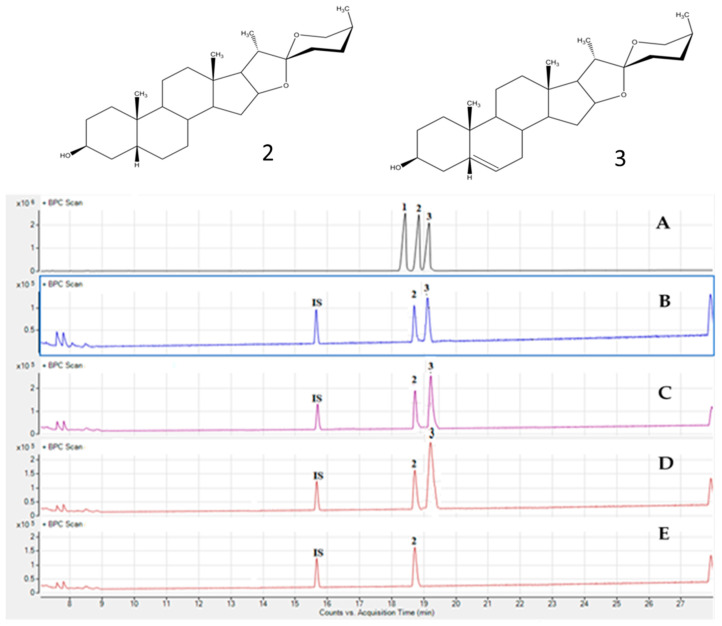
Chromatographic profile acquired by GC-MS of standards (**A**), stem (**B**), leaf (**C**), pericarp (**D**), and rhizome (**E**) of hydrolyzate crude saponins of *A. stipularis* and its sapogenins detected: 1: smilagenin, 2: sarsasapogenin, 3: diosgenin, IS: inter-standard (cholesterol).

The different detected saponins were subjected to hydrolysis, and the corresponding aglycones were analyzed through GC-MS. Figure 4 illustrates the identification of sarsasapogenin (Peak 2, M + 416 amu) and diosgenin (Peak 3, M + 414 amu) as aglycones in the hydrolysate of crude saponins from the leaf, stem, and pericarp of *A. stipularis*. This identification was established by comparing the retention time and mass spectrum with those of authentic standards.

The co-occurrence of sarsasapogenin and diosgenin was commonly observed in the aerial part of *A. officinalis* [32]. However, a singular peak corresponding to sarsasapogenin (Peak 2, M + 416 amu) was detected in the hydrolysate of crude saponins from the rhizome. Sarsasapogenin aglycone is frequently found in the roots of various Asparagus species, including *A. acutifolius* [7], *A. officinalis* [32,33], and *A. filicinus* [34].

The neutral sugars present in the saponin hydrolysates were also analyzed as alditol acetates by GC. Glucose (hexose) and xylose (pentose) were identified in HTSAP-1, STIPSAP-1, HTSAP-2, and STIPSAP-4, while xylose (pentose) and rhamnose (Deoxyhexose) were identified in STIPSAP-2, WSAP-4, and STIPSAP-3.

### 2.4. Quantification of Saponins in Asparagus stipularis Samples

Saponins were quantified according to methods outlined in the Materials and Methods, and the results showed that saponins were present in all the parts of the species investigated, but at varied levels (Table 5).

Saponins were most abundant in the underground parts and pericarp extracts, with content ranging from 56 g/kg DW to 54 g/kg DW, respectively. The saponin levels in the aerial parts (leaf and stem) of the plant were generally lower. As observed in Table 5, STIPSAP-2 and WSAP-4 were the most abundant saponins in all parts of *A. stipularis*, accounting for 60% and 30% in leaf and stem and 37% and 30% in pericarp, respectively, of the total saponins (Table 5). However, in the rhizome, only WSAP-4 was detected as the main saponin, constituting 58% of the total saponins. STIPSAP-3 was less abundant than STIPSAP-2 and WSAP-4 but showed considerable variation among individual samples in terms of both absolute content and relative composition of the total amount of saponins, being higher in pericarp (6.4 g/kg DW) and rhizome (4.5 g/kg DW) than in stem and leaf. The content of HTSAP-2 and STIPSAP-4 also varied significantly among individual samples, but their percentage in the total amount of saponins remained around 3–19%. They were detected in pericarp and rhizome but not in leaf and stem, where they were present in trace amounts. Rhizome exhibited a high content of HTSAP-2 (11 g/kg DW) and STIPSAP-4 (8 g/kg DW) compared to pericarps, which accounted for 6 g/kg DW for HTSAP-2 and 2 g/kg DW for STIPSAP-4. STIPSAP-1 was a minor constituent detected in pericarp and stem, representing 7% and 1% of the total saponins, respectively. A very low content of HTSAP-1 was only found in pericap, accounting for 1% of the total saponins.

### 2.5. Determination of Antioxidant Activities

**Figure 5:**
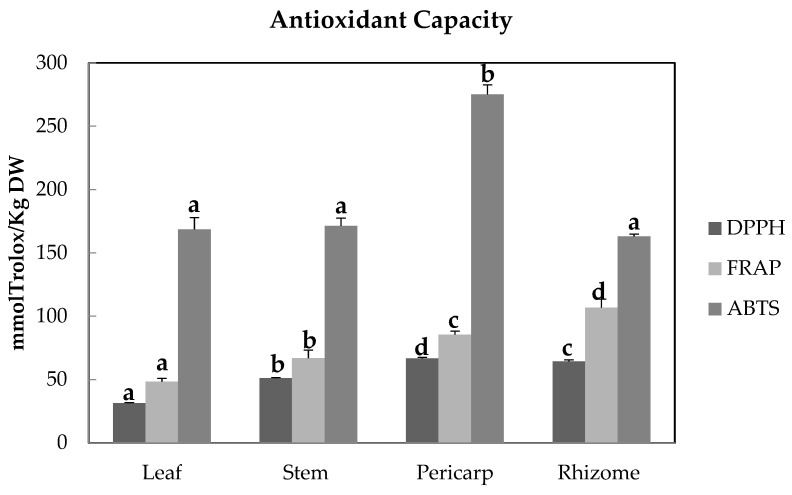
Antioxidant activities, expressed as mmolTrolox/Kg of dry weight in *A. stipularis* leaf, stem, pericarp, and rhizome ethanolic extracts, determined by DPPH, FRAP, and ABTS assays. DPPH: DPPH radical scavenging activity; FRAP: ferric reducing antioxidant power; ABTS: ABTS radical scavenging activity. Results correspond to the mean ± standard deviation of three replicates. Different letter(s) indicate the values are significantly different (*p* < 0.05). The comparison is for different samples with the same method.

DPPH, ABTS radical scavenging activity, and ferric reducing antioxidant power (FRAP) results (expressed as mmolTrolox/kg Dw values) are presented in Figure 5.

Overall, the pericarp and rhizome extracts exhibited high DPPH scavenging activity (67 and 64 mmol Trolox/kg DW, respectively), followed by the stem and leaf extracts (51 and 31 mmol Trolox/kg DW, respectively). In general, all extracts demonstrated ferric reducing power capability, with the following decreasing order: rhizome (107 mmol Trolox/kg DW), pericarp (86 mmol Trolox/kg DW), stem (67 mmol Trolox/kg DW), and leaf (48 mmol Trolox/kg DW) extracts. Concerning the ABTS^+^ radical assay, the radical-cation scavenging ability of ethanol extracts decreased in the following order: pericarp (275 mmol Trolox/kg DW), stem (171 mmol Trolox/kg DW), leaf (169 mmol Trolox/kg DW), and rhizome (163 mmol Trolox/kg DW).

The Trolox equivalence values determined for *A. stipularis* organ extracts indicated high antioxidant and free radical scavenging activities, especially in the rhizome and pericarp. Previous studies have evaluated the high antioxidant activity of extracts from *Asparagus* roots [35,36,37]. In comparison to our results, the antioxidant activity of root extracts from *A. racemosus*, a species highly sought after in the current international market, showed TEAC values under the DPPH, FRAP, and ABTS assays of 180 ± 2.56, 380 ± 4.85, and 55.80 ± 1.23 µmmol Trolox/100 g DW, respectively [38].

The abundant presence of bioactive compounds, such as saponins, phenolics, and flavonoids, detected in the organs of *A. stipularis* may be associated with the significant potential of these extracts as natural antioxidants. The antioxidant activity of many plant extracts is commonly attributed to the presence of saponins [39], flavonoids [40], and phenolics [41].

Pearson’s correlation analysis was conducted to assess the correlation between various antioxidant capacity assays and phytochemical contents in all samples (across the four organs). The antioxidant capacity, as measured by the DPPH and FRAP assays, showed positive correlations with total saponins (r = 0.911 and 0.917) and phenolics (r = 0.59 and 0.87), respectively (Table 6).

The correlation of antioxidant activities with phenolics aligns with numerous reports [42,43,44]. In various studies, including evaluations of eleven extracts of traditional Chinese antidiabetic plants [45], soybean [46], and shallot [39], a positive correlation between antioxidant activity and saponins was observed.

A notably significant negative correlation coefficient was observed for the total flavonoids content and antioxidant activity in this study. These findings align with those reported by Quiroga et al. [47], who observed correlation coefficients of r = −0.86 and r = −0.97 between the total flavonoids content and antioxidant activity, as determined by DPPH scavenging and the FRAP assay, respectively, in the essential oils of oregano and *Lippia turbinata*.

No significant correlation was found between ABTS scavenging activity and the determined metabolites in *A. stipularis* organ extracts.

Guillen et al. [48] proposed that the high antioxidant activity observed in asparagus, as determined by the DPPH free radical scavenging assay, is linked to the substantial quantity of phenolic compounds present in this plant. This aligns with the findings of the present study. The results indicated a positive correlation between saponins and phenolics with DPPH radical scavenging activity and ferric-reducing antioxidant power (FRAP) (Table 6), confirming the significance of these compounds in antioxidant activity.

### 2.6. Lipase Inhibitory Activity

**Figure 6:**
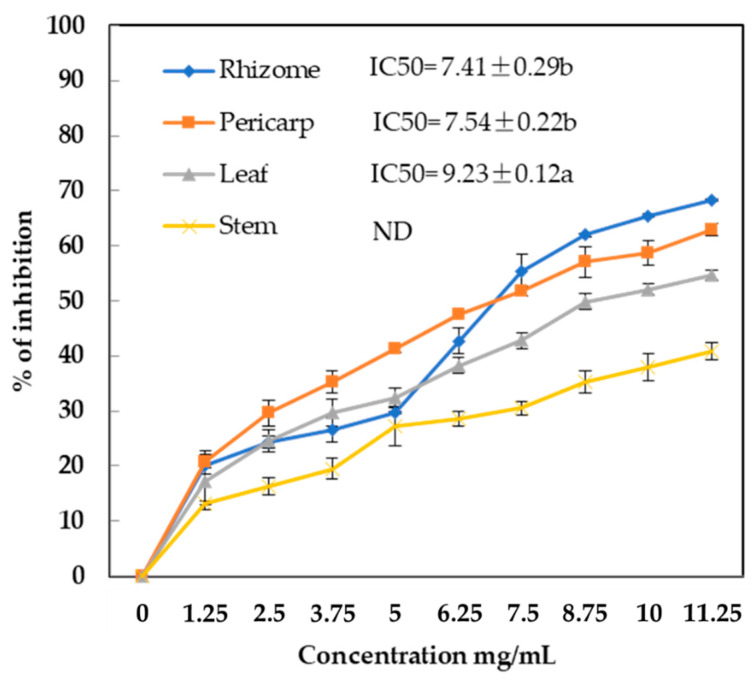
Pancreatic lipase inhibitory activity, expressed as IC50 (mg/mL) in leaf, pericarp, rhizome, and stem ethanolic extracts of *A. stipularis*. Results correspond to the mean ± standard deviation of three replicates. Different letter(s) indicate that the values are significantly different (*p* < 0.05). The comparison is for different samples with the same method. ND: not determined.

Pancreatic lipase (PL) plays an essential role in the hydrolysis of 50–70% of total dietary fats. Crude extracts of *A. stipularis* at different concentrations (0–11.25 mg/mL) have been examined for their PL inhibitory activity for the first time (Figure 6).

A dose-dependent pattern on pancreatic lipase inhibition activity was found for the ethanolic extracts from the leaf, stem, pericarp, and rhizome. At the highest dose, 11.25 mg/mL, rhizome, pericarp, leaf, and stem extracts inhibited pancreatic lipase activity by 68%, 63%, 55%, and 41%, respectively. Pericarp and rhizome extracts exhibited the best activity, with IC50 values of 7.41 ± 0.29 μg/mL and 7.54 ± 0.22 μg/mL, respectively, followed by leaf (9.23 ± 0.12 mg/mL). The activity of the stem extract was below 50% at the highest concentration assayed.

The results indicated that the inhibitory effects of *A. stipularis* pericarp and rhizome were strong compared to *Cyclocarea paliurus* extract, which exhibited pancreatic lipase inhibition in a dose-dependent manner with an IC50 value of 9.1 mg/mL [49]. The IC50 values obtained in the present study were similar to those reported by Conforti et al. [50], who analyzed 21 wild Mediterranean dietary plants and found IC50 values ranging from 5.48 to 10 mg/mL.

Saponins isolated from the root of *Platycodon grandiflorum* were shown to inhibit pancreatic lipase and have an antiobesity effect [51,52]. Various saponins isolated from *Platycodi Radix* seem to have been major compounds exerting lipase inhibitory action [53]. In addition, they found that saponin compounds of a relatively low molecular weight, such as platycodin D, deapio-platycodin D, and platycodin A, possess relative higher inhibiting activity than those compounds of a relatively high molecular weight (the latter possess one or two more glucose in structures than earlier) [54].

Saponins are the major compound in *A. stipularis* organs. It can be suggested that saponins might be a major contributor to lipase inhibition. Overall, the antilipase effect of the saponins of *A. stipularis* rhizome and pericarp suggests that they could be used as food additives to prevent obesity.

### 2.7. Cytotoxic Activity

**Figure 7:**
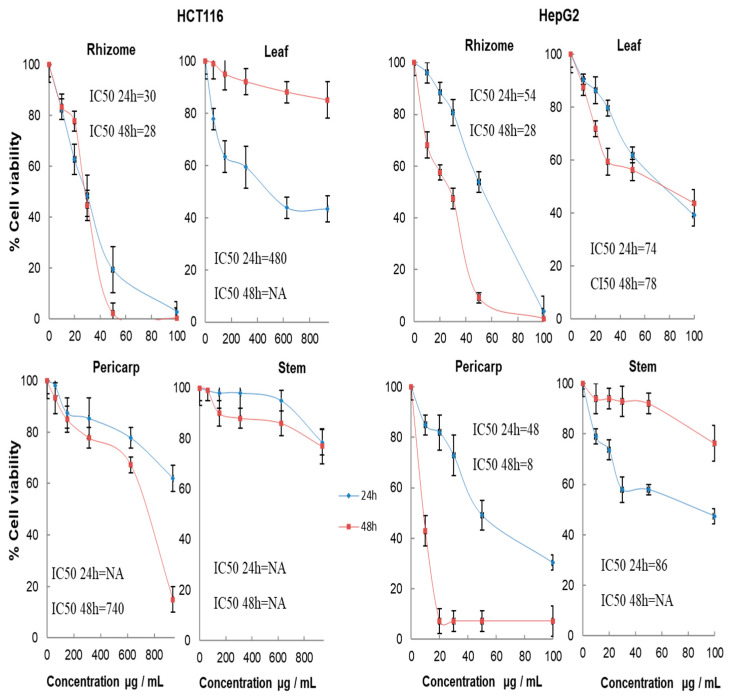
Cell viability assay on colon cancer (HCT-116) and on human hepatocellular carcinoma (HepG2) cells after 24 and 48 h incubation with serial concentrations of the different organ ethanolic extracts from *A. stipularis* rhizome, leaf, pericarp, and stem. The effect was measured by MTT cell viability assay, and the cytotoxic activities were expressed as IC50 μg/mL of dry weight. Results correspond to the mean ± standard deviation of four replicates.

In the present study, the cytotoxic activity of the ethanolic extracts of leaves, stems, pericarps, and rhizomes from *A. stipularis* against colon cancer (HCT-116) and liver cancer cell lines (HepG2) was determined by using the colorimetric MTT cytotoxicity assay at different concentrations. The time- and dose-dependent cytotoxic effects of different extracts on the growth of HCT-116 and HepG2 cell lines are summarized in Figure 7.

The rhizome extracts significantly decreased the percentage of viable cells in a concentration-dependent manner. At 50 µg/mL, the rhizome extracts inhibited 81% and 46% of HCT-116 and HepG2 cell lines after 24 h of treatment, and this inhibition increased to 98% and 91% after 48 h of treatments, respectively. At the highest concentration of 100 µg/mL, the viable HCT-116 and HepG2 cells were less than 5% after 24 h and 1% after 48 h of incubation. These results indicated that the rhizome extracts exhibited potent cytotoxic activity on the HCT-116 and HepG2 cell lines in the MTT assay. Concerning the leaf, stem, and pericarp extracts, at 100 µg/mL, after 24 h of incubation, those extracts inhibited, respectively, 61%, 53%, and 70% of HepG2 and 57%, 22%, and 38% of HCT-116. However, after 48 h of incubation, the percentage of the inhibition of HCT-116 and HepG2 was, respectively, 15% and 56% in leaf extracts, 23% and 24% in stem extracts, and 85% and 93% in pericarp extracts.

The effects of the extracts were expressed by IC50 values (concentration which inhibit 50% cell growth) as a parameter for cytotoxicity. The results indicate that the rhizome extract of *A. stipularis* is considered to be a particularly valuable source of effective antiproliferative and cytotoxic substances. Moreover, the IC50 values of the rhizome ethanolic extract for HCT-116 and HepG2 cells were 30 and 54 μg/mL after 24 and decreased to 28 μg/mL for both cell lines after 48 h of incubation.

The cytotoxic activity of leaf extracts against HCT-116 was much lower (IC50 values 480 μg/mL), and no activity was found in the stem and pericarp extracts after 24 h of treatment. After 48 h of treatment, no cytotoxic activity was found in the leaf and stem extracts, and the IC50 value of the pericarp extracts was 740 μg/mL. Concerning HepG2, great cytotoxic activity was found in the pericarp extracts, for which the IC50 value was 48 μg/mL after 24 h, and an improvement in the activity was found after 48 h of treatment, for which the IC50 value showed a significant decrease (8 μg/mL). Moreover, the cytotoxic activity of leaf extracts against HepG2 showed that the IC50 value was 75 μg/mL after 24 h and 48 h of incubation. However, in the stem extracts, the IC50 value was 86 μg/mL after 24 h of treatment, and no activity was found after 48 h. Our result showed that the rhizome extracts exhibited a potent cytotoxic activity against both of the cell lines studied. However, in the leaf, stem, and pericarp extracts, the activity was marked in the HepG2 rather than in the HCT-116.

The potent cytotoxic activity of the root extracts was also reported in other *Asparagus* species, such as *A. albus* (IC50 = 40 μg/mL against colon cancer cell lines HCT-116) [5], *A. racemosus* (IC50 = 100.5 µg/mL against non-small-cell lung cancer) [54], and *A. officinalis* (Asparanin A: IC50 = 6.20 µM after 24 h and 4 µM after 48 h against hepatocellular carcinoma HepG2 cells) [44]. In our previous study, the cytotoxic effect of the leaf and pericarps extracts of *A. albus* was also reported [5] as the fruit of *A. officinalis* [55,56] and the leaf of *A. racemosus* [56].

Previous studies have shown that saponins, which are naturally occurring compounds widely distributed in a variety of plants, exhibit anticancer activity by inducing cell cycle arrest and apoptosis, and by inhibiting proliferation in numerous types of human cancer cells [57,58,59].

## 3. Materials and Methods

### 3.1. Chemicals and Reagents

Authentic standards of quercetin (Q), kaempferol (K), isorhamnetin (IR), and rutin (quercetin-3-*O*-rutinoside); gallic acid, *p*-hydroxybenzoic acid, *p*-hydroxybenzaldehyde, vanillin, caffeic acid, *p*-hydroxyphenylacetic acid, ferulic acid, sinapic acid, and 2,2-diphenyl-1-picrylhydrazyl (DPPH free radical); ferric chloride, 2,2′-dipyridyl (99% minimum purity), Trolox (97% purity), porcine pancreatic lipase, *p*-nitrophenyl butyrate, methylthiazolyldiphenyl–tetrazolium bromide (MTT), ethanol, formic acid (96%), and acetonitrile (HPLC grade); and vanillic acid, *p*-coumaric acid, protocatechuic acid, syringic acid, sarsasapogenin, diosgenin, smilagenin, and trichloroacetic acid were purchased from Sigma-Aldrich Quimica (Madrid, Spain). Kaempferol-3-*O*-rutinoside (nicotiflorin), isorhamnetin 3-*O*-rutinoside (narcissin), and isorhamnetin 3-*O*-glucoside were purchased from Extrasynthese (Genay, France). Protodioscin (97%) and shatavarin (98.6%), purity checked by NMR, were purchased from Chromadex Chemical Co. (Barcelona, Spain). All cell culture reagents were purchased from Gibco (Madrid, Spain). All solvents were of HPLC grade purity (Romyl and Teknokroma, Barcelona, Spain). Ethanol, formic acid (96%), and acetonitrile, high-performance liquid chromatography (HPLC) grade, were purchased from Sigma Chemical Co. (St. Louis, MO, USA). The extraction solvents (ethyl acetate and methanol) were obtained from Romil Ltd. (Waterbeach, UK). Pure deionized water was obtained from a Milli-Q50 system (Millipore Corporation, Bedford, MA, USA).

### 3.2. Plant Material

*A. stipularis* L. plants were collected from Borj Cedria (22 km north Tunis). The plant materials were identified and authenticated by Mr. Mounir Kassri, and voucher specimens [PLM 53] were deposited at the Herbarium of the Department of Biology, Faculty of Science of Tunis, Tunisia. The samples of leaves, pericarps (fruits without seeds), and rhizomes (minimum 1 kg fresh weight each) of *A. stipularis* L. were cleaned and washed thoroughly with distilled water. Each organ was left at room temperature in the dark until dryness and then ground to fine powder in a Mettler AE 200 (Dangoumau type) grinder. All samples were stored at −20 °C until analysis.

### 3.3. Preparation of Ethanolic Extract

Ethanolic extraction was performed by mixing and homogenizing 1 g of plant material with 40 mL of ethanol/water (80:20, *v*/*v*) in an Ultraturrax (Ultra-Turrax T25, Janke & Kunkel/IKA Labortechnik IKA Works Spain, Sociedad Limitada, Barcelona ) for 1 min at maximum speed and then filtered through filter paper. The residue was extracted again in the same conditions. Ethanolic extracts were stored at −20 °C until analysis by HPLC. All extractions were made in duplicate.

### 3.4. Extraction of Phenolic Compounds

Free phenolics of the different plant part of *A. stipularis* were investigated. Extraction methods were adapted from Weidner et al. [60] and Sosulski et al. [61] with the following modifications: 20 mL of 80% ethanol extracts containing soluble phenolics, were concentrated to dryness by using a rotary evaporator at 35 °C, and 5 mL of distilled water was added. These aqueous solutions were adjusted to pH 2 and filtered through Whatman paper no. 1. The sample was extracted three times with ethyl acetate. The ethyl acetate phase was dried using a Savant Speed Vac (Bio101, Vista, CA, USA) and dissolved in 1 mL of methanol, HPLC grade. The final extracts were stored at −18 °C before analysis.

### 3.5. Analysis of Free Phenolics Composition by HPLC/MS

An HPLC Waters Alliance (Manchester, UK) system fitted to a Mediterranean Sea18 reverse-phase analytical column (25 cm length × 4.6 mm i.d., 5 μm particle size; Teknokroma, Barcelona, Spain) was used. An elution gradient was used with solvents A (water with 1% formic acid) and B (acetonitrile with 1% formic acid): the proportion of solvent B was increased from 0% to 15% in 10 min and then maintained at 15% for 5 min, then to 20% over the next 10 min, where it was maintained at 20% for 5, and to 100% over the next 5 min, where it was maintained at 100% B for 5 min, and then it finally returned to the initial conditions over the next 5 min. The column end was connected directly with a DAD (diode array detector) (Waters 996, Millipore, Manchester, UK) and, subsequently, the flow in the MS was regulated using a split (flow: 1 mL/min). The phenolics were detected using an online connected quadrupole mass analyzer (ZMD4, Micromass, Waters, Inc., Manchester, UK). Electron spray ionization (ESI) mass spectra were obtained at ionization energy of 70 eV, and the capillary voltage was 3 kV, desolvation temperature was 120 °C, source temperature was 80 °C, and extractor voltage was 12 V. The flow was maintained at 1 mL min^−1^, and the split ratio was 5:1 (UV detector MS) for each analysis.

Stock standard solutions of each compound (gallic acid, p-hydroxybenzoic acid, vanillic acid, p-hydroxybenzaldehyde, vanillin, caffeic acid, protocatechuic acid, syringic acid, p-hydroxyphenylacetic acid, p-coumaric acid, ferulic acid, and sinapic acid) were prepared by dissolving 10 mg of analytical standard in 10 mL of ethanol 80%. All solutions were stored at 4 °C. An intermediate solution containing all standard compounds (62.5 μg mL^−1^) was prepared in ethanol 80%, and dilutions from this solution were performed at different levels for calibration curves. Triplicate injections were made for each standard and sample. Individual compounds were identified by comparing the retention time and m/z values obtained by MS with those of standards obtained under the same conditions. The calibration curves used for quantitation peak areas were compared with the calibration curves generated by three repeated injections of known standards at seven concentrations (2.5–60 µg μL^−1^). Linearity ranges for calibration curves were determined.

### 3.6. Analysis and Quantification of Flavonoids by HPLC–DAD

A Jasco-LC-Net II ADC liquid chromatograph system equipped with a diode array detector (DAD) was used. Separation was carried out in a MEDITERRANEA SEA C18 reverse-phase analytical column (25 cm length × 4.6 mm i.d., 5 µm particle size; Teknokroma, Barcelona, Spain). A gradient of solvent A (water with 1% formic acid) and solvent B (acetonitrile with 1% formic acid) was used; the proportion of solvent B was increased from 0% to 20% in 20 min, then to 21% over the next 8 min, where it was maintained at 21% for 2 min, then to 30% over the next 10 min, and to 100% over the next 5 min, where it was maintained at 100% B for 5 min, and the initial conditions were finally returned to over the next 5 min. The flow rate was 1 mL/min, and the column temperature was 30 °C. Spectra from all peaks were recorded in the 200–600 nm range, and chromatograms were acquired at 360 nm for flavonoid glycosides and 370 nm for their aglycones.

For MS detection, a quadrupole mass analyzer (ZMD4, Micromass, Waters Inc., Manchester, UK) was used. Electrospray ionization (ESI) mass spectra were obtained at ionization energies of 50 and 100 eV (negative mode) and 50 eV (positive mode), with MS scans from m/z 100 to 1000. The capillary voltage was at 3 kV, desolvation temperature was 200 °C, source temperature was 100 °C, and extractor voltage was 12 V. The flow was diverted before the MS detector by means of a T connection, and flow through the detector was 0.3 mL/min.

The quantification of flavonoids was carried out as described by Fuentes-Alventosa et al. [22]. The identification of individual flavonoid glycosides was based on their retention times (Rts) and both spectroscopic and mass spectrometric data. The quantification of individual flavonoid monoglycosides and flavonoid diglycosides was directly performed by HPLC-DAD using an eight-point regression curve in the range of 0–250 μg on the basis of standards. Results were calculated from the means of the three replicates.

### 3.7. Acid Hydrolysis of Flavonoid Glycosides

Free flavonoid aglycones were released by acidic hydrolysis as follows: 2 and 5 g of freeze-dried material were extracted with 80 mL of 80% EtOH, as described above. In total, 20 mL of 6 M HCl was added, and the solution was incubated for 2 h, with constant mixing at 90 °C. The extract was filtered through filter paper and made up to 100 mL with 80% ethanol. The extracts were stored at −20 °C until analysis [23].

### 3.8. Analysis of Saponins

#### 3.8.1. Characterization and Quantification of Saponins by LC-MS

The evaluation of the saponin content was carried out as described by Vázquez-Castilla et al. [31]. An HPLC Waters Alliance (Manchester, UK) system fitted to a Mediterranean Sea C18 reverse-phase analytical column (25 cm length × 4.6 mm i.d., 5 μm particle size; Teknokroma, Barcelona, Spain) was used. An elution gradient was used with solvents A (water with 1% formic acid) and B (acetonitrile with 1% formic acid): 0–30 min, 20% B; 30–60 min, linear gradient to 30% B; 60 to 70 min, linear gradient to 100% B; and 70–80 min, linear gradient to 20% B. Saponins were detected using an online connected quadrupole mass analyzer (ZMD4, Micromass, Waters, Inc., Manchester, UK); the flow in the MS was regulated using a split (flow 0.3 mL/min). ESI mass spectra were obtained at ionization energies of 50 and 100 eV (negative mode) and 50 eV (positive mode) with scans from m/z 200 to 1200. The capillary voltage was 3 kV; the desolvation temperature was 200 °C; the source temperature was 100 °C; and the extractor voltage was 12 V.

Two different external standards were used: protodioscin and shatavarin IV. For each standard, 10 dilutions from 0 to 500 μg/mL were prepared and injected into the LC–MS system. For each standard, the selected ion chromatogram corresponding to its molecular ion in negative mode at 100 eV was integrated, and the peak area was plotted against the concentration and subjected to regression analysis.

#### 3.8.2. Determination of Exact Mass

For the determination of the exact mass, the saponin extract was injected in a high-resolution LC/MS system. The liquid chromatograph was Dionex Ultimate 3000 HPLC (Thermo Fisher Scientific, Waltham, MA, USA). Chromatographic separation was achieved in the same conditions than low-resolution LC-MS (previous section). A split post-column of 0.4 mL/min was introduced directly on the mass spectrometer ion source. Mass spectrometry was performed using a micrOTOF-QII High-Resolution Time-of-Flight mass spectrometer (UHR-TOF) with qQ-TOF geometry (Bruker Daltonics, Bremen, Germany) equipped with an ESI. The instrument was operated in negative-ion mode using a scan range of m/z 50–1200. Mass spectra were acquired through broad-band Collision-Induced Dissociation bbCID mode, providing MS and MS/MS spectra, simultaneously. The instrument control was performed using Bruker Daltonics HyStar 3.2 (Bruker bioscience España, Sevilla).

#### 3.8.3. Preparation of Saponin Extract and Purification of Individual Saponins

The dried ethanol extract of the different plant parts of *A. stipularis* was dissolved in water and loaded onto a column of Amberlite XAD-16. The column was washed with water, followed by 20%, 80%, and 96% ethanol, using a ratio of 1/4 (*v*/*v*) versus the loaded sample. The saponins were eluted with 80% ethanol. The saponin fraction was repetitively subjected to preparative scale reverse-phase HPLC on an ODS column (Shimadzu Prep K, 2.0 × 25.0 m) eluted with acetonitrile–water (1:1 *v*/*v*) at a flow rate of 20 mL/min.

#### 3.8.4. Hydrolysis of Saponins

A total of 100 µg of each purified saponin and 1 mg of total saponin fractions were separately treated with 2 mL of 1 M H_2_SO_4_ in 70% 2-propanol for 2 h at 100 °C. Cholesterol (50 µg) was added as internal standard. Then, 3 mL of water was added. Aglycones were extracted 3 times with 2 mL dichloromethane (CH_2_Cl_2_), and the organic solution was treated twice with 1 mL NH_4_OH 1M, and the solvent was then eliminated. The CH_2_Cl_2_ extracts were evaporated to dryness in a rotary evaporator. The obtained aglycones were dissolved in CH_2_Cl_2_ (200 µL) and analyzed by GC-MS.

#### 3.8.5. Analysis of Saponin Glycosidic Moiety

The glycosidic composition of the different saponins was determined by acid hydrolysis with 2 N trifluoroacetic acid (121 °C, 1 h) [62], derivatization to alditol acetates, and identification by gas chromatography [63].

An HP 6890 plus gas chromatograph (Hewlett-Packard, Palo Alto, CA, USA) fitted with a 30 m/250 μm/0.20 mm capillary column (SP-2330, Supelco, Bellefonte, PA, USA) was used. The carrier gas was helium with a constant flow equal to 2.2 mL/min and pressure of 21.5 psi (148.24 kPa). Injection was performed in splitless mode. The oven temperature was held at 50 °C for 2 min after injection and then programmed to 180 at 35 °C/min, held at 180 °C for 5 min, and then immediately increased to 220 at 5 °C/min, and then it was held at 220 °C for 22 min. Total run was 40.7 min. The injector temperature was 250 °C and flame ionization detector 300 °C. Neutral sugars L-rhamnose, D-fucose, L-arabinose, D-xylose, D-mannose, D-galactose, and D-glucose were identified. Myo-inositol was used as the internal standard.

### 3.9. Determination of the Antioxidant Capacity

Method of 2,2-diphenyl-1-picrylhydrazyl (DPPH) [64]: 10 µL of extracts was added to 190 µL of DPPH^•^ (3.8 mg/50 mL methanol); after 30 min in obscurity, the absorbance was measured (in triplicate) at 480 nm. The efficient concentration, EC50, which represents the amount of antioxidant necessary to decrease the initial absorbance by 50%, was calculated from a calibration curve by linear regression for each sample. The activity was expressed as millimoles of Trolox equivalent antioxidant capacity (TEAC) per kilogram of dry sample by means of a dose–response curve for Trolox.

Method of 2,2-azino-bis(3 ethylbenzothiazoline-6-sulphonic acid) (ABTS): the ABTS assay was performed according to the method of Gonçalves et al. [65], with some modifications [66]. Aliquots of 13 μL of each extract and their dilutions were added to 187 μL of the ABTS^•+^ solution in a 96-well microplate in triplicate. For each sample, a blank with ethanol instead of ABTS^•+^ solution was included. A delay of 30 min was programmed into the reader before readings at 414, 655, and 750 nm. The results were expressed in terms of millimoles of Trolox equivalent antioxidant capacity (TEAC) per kilogram of dry sample by means of a dose–response curve for Trolox.

Determination of ferric reducing antioxidant power: A modification of Psarra et al.’s [67] method was used. FeCl_3_ was employed as oxidant. Fe^2+^ ion produced from the redox reaction forms a colored product with 2,2′-dipyridyl. For the determination of the reducing power of extracts, a microplate reader was used. In total, 10 μL of each extract and their dilutions, and 10 μL of 6 mM FeCl_3_ in 5 mM citric acid, were placed in each microplate well in quadruplicate. For each sample, a blank without FeCl_3_ was included. After dosification, the microplate was incubated for 20 min at 50 °C in an oven. Following this, 180 μL of 5 g/L dipyridyl solution in 1.2% trichloroacetic acid was added to each well. Afterward, a delay of 30 min was programmed in the reader before reading at 490 nm. The results were expressed as millimoles of Trolox equivalent antioxidant capacity (TEAC) per kilogram of dry sample by means of a dose–response curve for Trolox.

### 3.10. Pancreatic Lipase Inhibitory Activity Assay In Vitro

Lipase activity was measured using p-NPB as a substrate. This method was modified by one previously described by Kim et al. [68]. Briefly, an enzyme buffer was prepared by the addition 20 μL of solution of porcine pancreatic lipase (20 mg/mL in TRIS buffer, pH 7) to 160 μL of Tris buffer (100 mmol/L Tris–HCl and 5 mmol/L CaCl_2_, pH 7.0). Then, increasing concentrations of various extracts (ranging from 0 to 6.3 mg/mL) dissolved in TRIS buffer were mixed with 20 μL of the enzyme buffer and incubated for 30 min at 37 °C. Twenty microliters of substrate (10 mmol/L p-NPB in dimethyl formamide) was then added. Lipase activity was determined by measuring the hydrolysis of p-NPB to p-nitrophenol at 405 nm using an ELISA reader. The inhibition of lipase activity was expressed as the percentage decrease in OD when porcine pancreatic lipase was incubated with the test compounds. Lipase inhibition (%) was calculated according to the following formula:

Inhibitory activity (I %) = (A − B)/A × 100

where A is lipase activity in the reaction solution without sample and B is lipase activity in the reaction solution containing sample. The measurements were made in triplicate, and the IC50 (inhibitory concentration at which 50% inhibition of enzyme activity occurs) values of the test samples were determined by performing the assay as above with varying concentrations of the test samples. The IC50 values were determined from the plots of the percentage inhibition Vs concentration.

### 3.11. Cytotoxicity against Cancer Cell Lines

Two cancer cell lines were included in the present study. Human colon cancer HCT-116 and human liver cancer HepG2 cell lines were purchased from the American Type Culture Collection (ATCC, Manassas, VA, USA). Cell culture media were as follows: HCT-116: McCoy’s 5A medium supplemented with 10% heat-inactivated fetal bovine serum (FBS), 100 U/mL penicillin, and 100 µg/mL streptomycin; HepG2: Eagle’s Minimum Essential Medium supplemented with 10% heat-inactivated FBS, 100 U/mL penicillin, and 100 µg/mL streptomycin. All cell lines were cultured at 37 °C and 5% CO_2_ incubators.

Cell viability was assayed based on the ability of live cells to reduce MTT. HCT-116 human colon cancer cells were cultured in 96-well plates in triplicate at a density of 10^4^ cells/well in 200 μL of medium. The cells were grown to 70–80% confluence and then treated with increasing concentrations of plant extracts (ranging from 0 to 600 μg/mL) dissolved in water.

Cells were incubated for 24 h and 48 h, and then 20 μL of the MTT solution (5 mg/mL in phosphate-buffered saline) was added to each well. Cells were incubated for 3 h at 37 °C in a humidified chamber with 5% CO_2_. The supernatant was then sucked off, after which 100 μL of DMSO was added to each well. The culture plate was gently agitated (about 30 min) to make purple formazan dissolve completely. The concentration of formazan was measured at 490 nm using a Multiskan Spectrum microplate reader (ThermoLabsystems, Waltham, MA, USA). The IC50 values were calculated from the regression curves of % cell viability vs. extract concentration for each assay. All MTT assays were carried out in three separate trials, with each experiment including five replicate sets.

### 3.12. Statistical Analysis

All data presented are the mean ± standard errors from three measurements. Means comparison were determined by a one-way ANOVA, followed by the “Duncan Test”. Differences were considered significant at *p* < 0.05. Statistic, 7.0 package was used.

## 4. Conclusions

The present study focused on the antioxidant, pancreatic lipase inhibitor, and cytotoxic potential of different plant parts of *A. stipularis.* Additionally, this study aimed to determine the chemical composition of these plant parts, specifically focusing on phenolic, flavonoid, and saponin compounds. The obtained extracts possess significant antioxidant and cytotoxicity capacities in vitro, as well as moderate pancreatic lipase inhibitory activity. The findings of this study revealed that *A. stipularis* extracts could be used as a readily accessible source of natural antioxidants, with potential application as a crude material in the pharmaceutical industry.

## Figures and Tables

**Table 1:** Simple phenolics and phenolic acids composition of ethanolic extracts from *A. stipularis*.

Phenolics	Leaf	Stem	Pericarp	Rhizome
Gallic acid	n.d.	35.48 ± 0.12 a	36.85 ± 0.79 a	61.72 ± 0.71 b
Protocatechuic acid	14.12 ± 0.64 a	13.70 ± 0.27 a	173.66 ± 0.80 c	105.97 ± 0.11 b
p-Hydroxybenzoic acid	23.30 ± 0.34 c	13.75 ± 0.37 a	20.83 ± 0.41 b	160.85 ± 0.54 d
Vanillic acid	6.72 ± 0.37 a	n.d.	n.d.	84.75 ± 0.65 b
Caffeic acid	32.70 ± 1.13 b	93.06 ± 0.22 d	2.89 ± 0.17 a	78.09 ± 0.20 c
OH-benzaldehyde	12.15 ± 1.03 b	10.25 ± 0.18 a	10.10 ± 0.37 a	39.76 ± 0.16 c
Syringic acid	n.d.	n.d.	n.d.	81.20 ± 0.46
Vanillin	n.d.	20.86 ± 0.34 b	n.d.	15.00 ± 0.34 a
p-Coumaric acid	8.27 ± 0.21 c	2.79 ± 0.13 a	7.66 ± 0.01 b	133.25 ± 0.01 d
t-ferulic acid	25.00 ± 0.27 a	28.82 ± 0.38 b	24.60 ± 0.76 a	156.99 ± 0.34 c
t-Sinapic acid	n.d.	n.d.	12.94 ± 0.28 b	12.66 ± 0.76 a
Total	122.27 ± 0.97 a	218.70 ± 0.90 b	289.53 ± 1.99 c	930.23 ± 4.27 d

Data, expressed as mg/Kg dry samples from leaf, stem, pericarp, and rhizome, are the mean of three replicates. Different letters within the same row mean that there are significant differences *(p* < 0.05). nd, not detected.

**Table 2:** Peak numbers, retention times (Rts), UV, and main ion species observed during HPLC-DAD-MS analysis and assigned structures of the flavonoids from different plant parts of *A. stipularis*.

Peak #	Rt (min)	λmax (nm)	Molecular Ion [M + H]^+^ (m/z)	Ions (ESI+) (m/z)	Assigned Structures
1	19.10	256, 308sh, 352	919	757,611,465,303	Quercetin-3-glucosyl-(1->4)-rhamnoside-7-rutinoside
2	20.40	252, 288sh, 352	933	771,625,479,317	isorhamnetin-3-glucosyl-(1->4)-rhamnoside-7-rutinoside
3	25.30	255, 267sh, 352	757	611,465,303	Quercetin-3-*O*-rhamnosylrutinoside
4	27.10	256, 267sh, 354	773	627,481,303	Quercetin-3-*O*-glucosyl-rutinoside
5	27.50	254, 268sh, 355	771	625,479,317	Isorhamnetin-3-*O*-rhamnosyl-rutinoside
6	29.70	255, 267sh, 355	611	465,303	Quercetin-3-*O*-rhamnoglucoside (rutin)
7	41.45	252, 267sh, 372	303	-	Quercetin
8	42.36	257, 288	273	-	Naringenin
9	43.58	264, 296sh, 364	287	-	Kaempferol

**Table 3:** Quantification of the flavonoids found in the different plant part of *A. stipularis* extracts.

Peak	Flavonoids Compound	Quantification (mg/kg, dw)			
		Leaf	Stem	Pericarp	Rhizome
1	Q-3-glucosyl-rhamnosyl-rutinoside	519 ± 48.12 b	236 ± 5 a	n.d.	n.d.
2	IR-3-glucosyl-rhamnosyl-rutinoside	1343 ± 92.88 b	570 ± 38 a	n.d.	n.d.
3	Q-3-rhamnosylrutinoside	621 ± 60 c	250 ± 3 a	370 ± 25 b	n.d.
4	Q-3-glucosyl-rutinoside	296 ± 18 a	1095 ± 41 b	n.d.	n.d.
5	IR-3-rhamnosyl-rutinoside	619 ± 40	n.d.	n.d.	n.d.
6	Rutin	337 ± 27 a	n.d.	925 ± 72 b	n.d.
7	Quercetin	n.d.	n.d.	43.98 ± 0.33 c	n.d
8	Naringenin	5.06 ± 0.21 a	6.18 ± 0.21 b	6.24 ± 0.44 b	n.d.
9	Kaempferol	47.78 ± 0.65 b	33.80 ± 1.75 a	69.93 ± 0.07 c	n.d.
	Free flavonoids	52.84 ± 0.86 c	39.98 ± 1.96 b	120.15 ± 0.70 d	n.d
	Total flavonoids glycoside	3734 ± 287 c	2151 ± 88 b	1598 ± 94 a	n.d.

Data mg/kg dry leaf, stem, pericarp, and rhizome are the mean of three replicates. Different letters within the same row mean that there are significant differences (*p* < 0.05). n.d. non detected.

**Table 4:** Characterization of the saponins tentatively identified by UHPLC/Q-TOF-MS (+/−) of the ethanolic extracts of different organs from *A. stipularis*.

			Molecular Ion (m/z)			Ion Fragmentation	
**saponins**	Rt ^a^ (min)	Formula	[M − H]^−^ Experimental	[M − H]^−^ Theoretical	Error (ppm)	Neg mode	Pos mode
**HTSAP-1**	24.57	C_50_H_83_O_23_	1051.5331	1051.5311	−1.8	Pen919-Hex757-Hex595-Hex433 Hex889-Pen757-Hex595-Hex433	[-Na-H_2_O]1035-Hex873-Hex711-Pen579-Hex417 [-Na-H_2_O]1035-Hex873-Pen741-Hex579-Hex417
**STIPSAP-1**	28.29	C_49_H_79_O_22_	1019.5068	1019.5050	1.8	^b^ Pen887-Pen755-^c^ Hex593-Hex431	[-Na-H_2_O]1003-Pen871-Hex709-Pen577-Hex415
**HTSAP-2**	29.37	C_49_H_81_O_22_	1021.5225	1021.5210	−1.5	Pen889-Pen757-Hex595-Hex433	[-Na-H_2_O]1005-Pen873-Hex711-Pen579-Hex417
**STIPSAP-2**	37.12	C_35_H_63_O_22_	835.3816	835.3816	−0.1	nd	[-Na-H_2_O]819-Hex657-^d^ DoHex511-^e^ Unk415
**WSAP-4**	37.59	C_35_H_65_O_22_	837.3997	837.3973	1.4	nd	[-Na-H_2_O]821-Hex659-DoHex513-Unk417
**STIPSAP-3**	43.01	C_35_H_63_O_22_	835.3792	835.3816	3.0	nd	[-Na-H_2_O]819-Hex657-DoHex511-Unk415
**STIPSAP-4**	48.75	C_43_H_69_O_16_	841.4591	841.4614	2.7	Pen709-Pen577-Unk433	[-Na-H_2_O]825-Pen693-Pen561-Unk 417

^a^ Rt = retention time. ^b^ Pen = pentose. ^c^ Hex = hexose. ^d^ DoHex = deoxyhexose. ^e^ Unk = unknown. Genins = 431, 433, 415, and 417.

**Table 5:** Saponins identified in leaf, stem, pericarp, and rhizome ethanolic extracts of *A. stipularis* calculated from the molecular ion of the peak area obtained by HPLC-MS.

Saponin	Leaf	Stem	Pericarp	Rhizome
**HTSAP-1**	-	-	411.8 ± 10.3 1%	-
**STIPSAP-1**	-	179.8 ± 3.5 1%	3844.2 ± 340.6 7%	-
**HTSAP-2**	-	-	5640.3 ± 439.6 10%	10,772.3 ± 31.9 19%
**STIPSAP-2**	12,656.0 ± 964.6 60%	16,367.8 ± 1048.6 60%	19,730.2 ± 948.1 37%	-
**WSAP-4**	6937.7 ± 470.3 33%	7727.0 ± 664.0 28%	16,374.2 ± 997.2 30%	32,245.9 ± 142.3 58%
**STIPSAP-3**	1994.9 ± 135.6 9%	3171.6 ± 275.2 12%	6383.2 ± 195.5 12%	4540.8 ± 483.5 8%
**STIPSAP-4**	-	-	1638.3 ± 94.8 3%	8128.6 ± 582.1 15%
**Total** **saponins**	21,588.6 ± 1570.5 d	27,446.3 ± 1984.3 c	54,022.2 ± 1129.9 b	55,687.6 ± 209.0 a

Data mg/Kg dry leaf, stem, pericarp, and rhizome are the mean of three replicates. Different letters within the same row mean that there are significant differences (*p* < 0.05).

**Table 6:** Pearson’s correlation coefficients of total phenolics (TPs), flavonoids (TFs), and saponins (TSs) contents, and antioxidant capacities determined by ABTS, DPPH and FRAP assays in ethanolic extracts of different plant part of *A. stipularis*.

	DPPH	FRAP	ABTS
**TPs**	** 0.586826	*** 0.868038	−0.253
**TFs**	*** 0.861817	*** −0.966198	−0.055
**TSs**	*** 0.910861	*** 0.916607	0.488

** Correlation is significant at the 0.05 level. *** Correlation is significant at the 0.01 level.

## Data Availability

The data presented in this study are available in the article.
